# Magnetic Resonance Imaging as a Diagnostic Tool for Ilio-Femoro-Caval Deep Venous Thrombosis

**DOI:** 10.3390/jimaging10030066

**Published:** 2024-03-08

**Authors:** Lisbeth Lyhne, Kim Christian Houlind, Johnny Christensen, Radu L. Vijdea, Meinhard R. Hansen, Malene Roland V. Pedersen, Helle Precht

**Affiliations:** 1 Department of Radiology, Kolding, Lillebaelt Hospital, University Hospitals of Southern Denmark, Sygehusvej 24, 6000 Kolding, Denmarkradu.lucian.vijdea@rsyd.dk (R.L.V.);; 2 Department of Vascular Surgery Kolding, Lillebaelt Hospital, University Hospitals of Southern Denmark, Sygehusvej 24, 6000 Kolding, Denmark; kim.christian.houlind@rsyd.dk; 3 Department of Regional Health Research, University of Southern Denmark, J.B. Winsløws Vej 19, 3, 5000 Odense C, Denmark; 4 Department of Radiology, Vejle Hospital, University Hospital of Southern Denmark, Beriderbakken 4, 7100 Vejle, Denmark; 5 Health Sciences Research Centre, UCL University College, Niels Bohrs Allé 1, 5230 Odense M, Denmark

**Keywords:** deep venous thrombosis, magnetic resonance phlebography, digital subtraction phlebography

## Abstract

This study aimed to test the accuracy of a magnetic resonance imaging (MRI)-based method to detect and characterise deep venous thrombosis (DVT) in the ilio-femoro-caval veins. Patients with verified DVT in the lower extremities with extension of the thrombi to the iliac veins, who were suitable for catheter-based venous thrombolysis, were included in this study. Before the intervention, magnetic resonance venography (MRV) was performed, and the ilio-femoro-caval veins were independently evaluated for normal appearance, stenosis, and occlusion by two single-blinded observers. The same procedure was used to evaluate digital subtraction phlebography (DSP), considered to be the gold standard, which made it possible to compare the results. A total of 123 patients were included for MRV and DSP, resulting in 246 image sets to be analysed. In total, 496 segments were analysed for occlusion, stenosis, or normal appearance. The highest sensitivity compared occlusion with either normal or stenosis (0.98) in MRV, while the lowest was found between stenosis and normal (0.84). Specificity varied from 0.59 (stenosis >< occlusion) to 0.94 (occlusion >< normal). The Kappa statistic was calculated as a measure of inter-observer agreement. The kappa value for MRV was 0.91 and for DSP, 0.80. In conclusion, MRV represents a sensitive method to analyse DVT in the pelvis veins with advantages such as no radiation and contrast and the possibility to investigate the anatomical relationship in the area.

## 1. Introduction

Acute deep venous thrombosis (DVT) in the ilio-femoro-caval veins is an acute condition that requires fast and accurate detection and immediate treatment to avoid significant morbidity and mortality [1,2,3,4]. The typical clinical symptoms of DVT are pain in one leg, usually in the calf when walking or standing, and swelling in one leg as warm and red skin around the area in pain [3]. Ultrasound is the first choice of investigation when patients present with clinical symptoms of DVT in the extremities. The advantages of ultrasound examination are that it is readily available, quick, reliable, cheap, non-invasive, devoid of ionising radiation, and unnecessary for intravenous contrast. It can, however, be challenging to use as a diagnostic tool for the deep veins of the abdomen and pelvis if the patient is obese, has air-filled intestines, or if the symptoms are caused by extrinsic compression of a vein by a pelvis mass or other perivascular pathology [2,5,6]. This limitation may be of minor significance when deciding whether to initiate oral or systemic anti-thrombotic therapy. However, when catheter-directed thrombolysis is considered, the information on whether and to what extent the thrombus is present above the inguinal ligament is essential because the correct indication is necessary, and the intervention must be planned so that the execution has the best prerequisites [6,7,8].

Contrast venography, first described in 1963 [9], was the first imaging procedure to diagnose DVT and is still considered the gold standard [2,10,11]. Though the procedure is good at visualising the deep veins of the abdomen and pelvis, it is invasive with the risks connected to this, requires expertise, requires a large volume of contrast, and involves ionising radiation. Currently, it is seldom used because newer and safer techniques like contrast-enhanced computerised tomography venography (CTV) and magnetic resonance venography (MRV) have evolved. Contrast venography is still used when concurrent interventions are planned [2,6,12].

The advantages of CTV are that it is cheap, quick, widely available, provides anatomical detail, accurate thrombus detection, and is a non-invasive procedure, but negatively, the patients will still be exposed to ionising radiation and contrast [2,6,12,13].

MRV is good at providing anatomical details and accurate thrombus detection. The procedure is non-invasive, contrast is unnecessary, and ionising radiation is not used. The disadvantages of MRV are low availability, the long duration of the examination, the interpretation requires expertise and patients with MR-unsafe implants cannot be scanned [2,6,14]. Despite this, the advantages seem to outweigh the disadvantages, especially when considering that a considerable number of patients with DVT in the ilio-femoro-caval veins are young [15].

Different imaging possibilities for diagnosing DVT have been compared over recent years [1,2,4,6,8,9,10,11,12,14,15,16]. Typical studies compare the gold standard of catheter-directed thrombolysis with either CTV, ultrasound, or MRV. An elder study by Carpenter et al. compared the diagnostic outcome of contrast venography with MRV, and a few patients also have duplex scanning for diagnosing their DVT. It was concluded that MRV is an accurate, non-invasive venographic technique for detecting DVT, although the method behind the included modalities has significantly changed since 1993 [3]. A newer study by Aschauer et al. examined 88 patients presenting clinical signs of deep vein thrombosis and/or pulmonary artery embolism of various stages. Excellent MR Angiography image quality was found in 87%, and a diagnostic image quality of MRV in 90% was observed, although not compared by diagnostic accuracy to other imaging modalities [4]. Not all evidence supports the replacement in favour of MRV, as Abdalla et al., in a systematic review, show a preference for an ultrasound to diagnose DVT, although suggesting MRV for obese patients [16]. To our knowledge, former studies did include a limited number of patients (88 patients maximum) with only a few artery segments evaluated by two observers and often focused on both DVT and pulmonary artery embolism [1,2,4,6,8,9,10,11,12,14,15,16].

This study aimed to test the accuracy of a non-invasive MRV technique for detecting and characterising ilio-femoro-caval DVT compared to the gold standard when catheter-directed thrombolysis is considered.

## 2. Materials and Methods

### 2.1. Patients

At our institution, we perform catheter-directed thrombolysis in patients with ilio-femoro-caval DVT [15]. In cases when patients are referred from other hospitals, the diagnosis is made using CT and confirmed by MRI upon arrival at our institution before catheter-directed thrombolysis is performed. Patients who presented directly with ilio-femoro-caval DVT at our institution have their diagnosis made using MRI before catheter-directed thrombolysis is performed. Exact knowledge concerning the extent of the thrombosis is essential in our decision on catheter-directed thrombolysis treatment before the patients are referred to our institution from other hospitals. The distal extent of the thrombosis was not crucial in this setting because even though the popliteal vein is occluded, catheter-directed thrombolysis often has a systemic effect and dissolves thrombosis distal for the puncture place. The decision to offer catheter-directed thrombolysis is made in a multi-disciplinary conference, including vascular surgeons and interventional radiologists.

The inclusion criteria for catheter-based vein thrombolysis are as follows:

− Patients with verified iliac and femoral DVT and pronounced symptoms;
− Between 15 and 75 years of age;
− Duration of symptoms <14 days.

Patients were excluded from this study if they were not eligible for magnetic resonance imaging (MRI), had previous ipsilateral DVT, were diagnosed with cancer, or had an expected lifespan of <2 years. The reason for excluding patients diagnosed with cancer or having an expected lifespan of less than two years is that the exclusion criteria are for being offered treatment and part of the retrospective study. The reason for excluding patients with a short expected lifespan for catheter-directed thrombolysis is that the objective of the treatment is to avoid developing catheter-directed thrombolysis in the long run after a DVT. Hence, patients with short life expectancy will only have the risks and not the benefits of the treatment. The patient was also excluded if the MRV findings suggested by diffusion sequence that the DVT was older than 14 days.

A magnetic resonance imaging (MRI) scan of the pelvis and abdomen was performed for all vein thrombolysis candidates on the day of hospitalisation. Contrast phlebography was used when catheter-based vein thrombolysis was performed. Therefore, all patients received both an MRI scan and a contrast phlebography.

### 2.2. MRI Technique

The non-contrast MRI scans of the abdomen and the groins were performed by a Philips Inginia 1.5 T (Philips Healthcare, Best, the Netherlands), including a protocol of balanced fast field echo (BFFE) sequences, heart frequency controlled, as shown in Table 1.

The BFFE sequences are advantageous as a dynamic examination of the motility of the heart. Still, they are also helpful for a stationary depiction of records of, e.g., abdominal arteries and veins.

**Figure 1:**
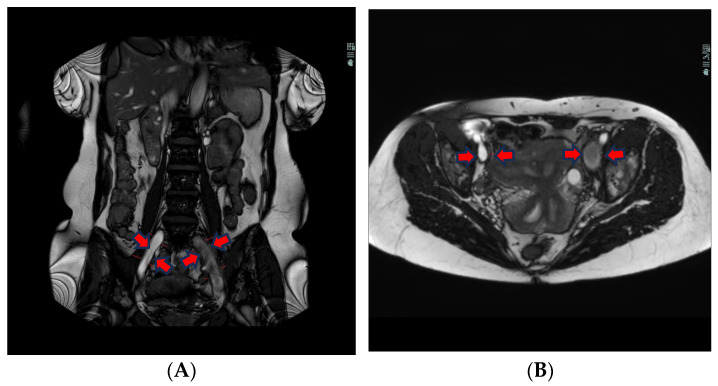
(**A**) Coronal BFFE shows a hypointense signal and a bit ecstatic thrombosed left external iliac vein. The right iliac vein has a hyperintense signal and is patent. (**B**) Transversal BFFE shows ecstatic and thrombosed left iliac vein. The right iliac vein has a hyperintense signal and is patent.

The liquid blood in veins and arteries appears white, whereas solidified blood, as in vein thrombi, is dark, spanning from grey to black (Figure 1A,B).

**Figure 2:**
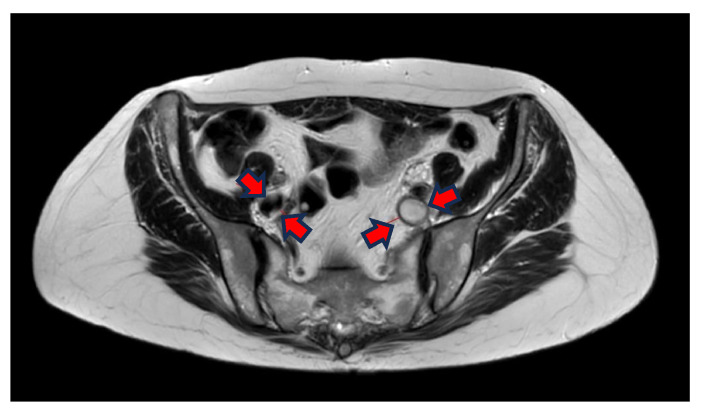
Transversal T2W imaging shows that the thrombus in the left iliac vein has a hyperintense signal because of clotted blood in the thrombi and lack of flow in the vein. The right iliac vein has a hypointense signal because the flowing blood = flow void.

The T2-weighted (T2w) sequence shows, on the other hand, liquid blood as black and signal empty, i.e., flow void. This is due to the protons in the blood moving during the period between the excitation pulse and the measured pulse, meaning that there is not a null signal from the area where the protons were at the excitation. The protons in thrombi, on the other hand, are “locked” in the coagulated stagnant blood and, therefore, indicate a coagulated liquid, which in turn is white on a T2w sequence (Figure 2).

The T2w sequence is less sensitive to artefacts than the BFFE, which can be extremely important for patients having more DVT or being treated with stents earlier. The T2w has a better linear resolution and gives better tissue differentiation in the images (see differences between Figure 1 and Figure 2).

The scans have recently been supplemented with diffusion-weighted imaging (DWI) sequences, which show pelvic thrombi as being white on the DWI b800 images and a 3D image of the extent of the thrombi. If there is no diffusion restriction, the DVT is older than 2–3 weeks, and, as described (in Section 2.1), the patient was excluded from this study.

### 2.3. Catheter-Based Digital Subtraction Phlebography

Digital subtraction phlebography (DSP) was performed in all patients using the same monoplane Angiography System (Siemens Artis Q.zen, Siemens Healthineers, Erlangen, Germany), using a manual contrast injection of 20 mL of Visipaque 270^®^ (General Electric Health Care, Milwaukee, WI, USA) for each subtracted series. The injections were performed systematically, first using a 6 F sheath (Prelude PRO™, Merit Medical, South Jordan, UT, USA) inserted in the popliteal vein, and, afterward, utilising a 4 F, 90 cm long, straight open-end and side-holes diagnostic catheter (NYLEX™ Angiographic Catheter, Cordis, FL, USA) first placed in the common femoral vein, followed by the external iliac vein and in the common iliac vein. DSP dynamic series were recorded and stored in our department’s picture archiving communication system (PACS).

### 2.4. Image Evaluation

**Figure 3:**
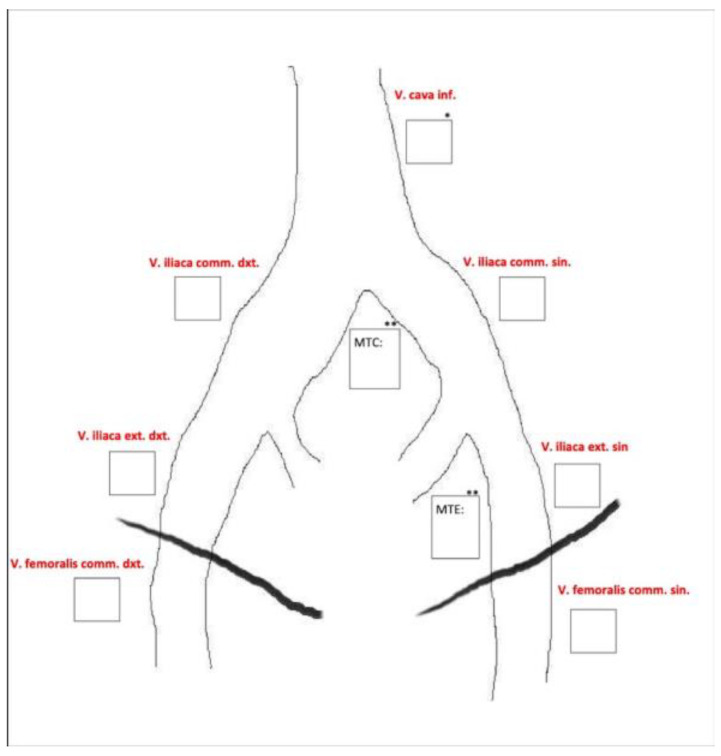
Predefined data sheet used for image evaluation.

MRV was performed on all patients within two weeks before the placement of the thrombolysis catheter. DSP was conducted in the angiography suite at the start of the catheter-directed thrombolysis. Two experienced radiologists assessed each examination segment according to a predefined data sheet (Figure 3). All image sets were evaluated using a diagnostic monitor in a reporting room with optimal light settings, and the radiologists were allowed to work undisturbed.

Initially, each observer was blinded to the other’s assessment. If the two observers disagreed, a consensus interpretation of the imaging was made, and the findings were reported as if only one observer had interpreted the images.

The extension of the thrombi was assessed, and whether it was a full- or a partial thrombosis was evaluated. An assessment of the compression phenomenon of the proximal part of the common iliac veins, known as May–Thurner’s syndrome, and cava vein atresia were also recorded when deemed relevant. Patients with the compression phenomenon of the proximal part of the common iliac vein often need a stent in extension to the thrombolytic treatment. This can be planned before the intervention when change is caught at the MRV.

### 2.5. Statistical Analyses

The sensitivity, specificity, and accuracy were calculated for patent vessels (0), stenosis (1), and occlusion (2) for each of the techniques, MRV and DSP. To assess inter-observer agreement, the kappa value (κ) was calculated. Where the observers are in perfect agreement, then κ = 1. Where there is no agreement among the observers other than expected by chance, κ = 0.

## 3. Results

A total of 123 patients received both MRV and DSP, resulting in 246 image sets to be analysed by two observers, both experienced in interventional radiology in peripheral vascular disease between 10 and 27 years of experience, respectively. In total, 496 segments were analysed for occlusion, stenosis (partial thrombosis), or patent vein (no thrombosis) at seven different anatomical landmarks cf. Figure 1.

The 123 patients consisted of 80 (65%) women and 43 (35%) men, all having DVT; the youngest was 15, and the oldest was 75 years old. The majority of the patients with DVT in the pelvic veins were young women; see Table 2.

The sensitivity, specificity, and positive and negative predictive values for MRV compared with DSP in the four scenarios described below are shown in Table 3.

Comparing patient veins with thrombosed veins (stenosis and occlusion), 387 segments (78%) showed signs of stenosis or occlusion in both modalities. Nineteen segments (4%) showed stenosis/occlusion on the MRV but normal signs on the DSP. Thirty segments (6%) showed normal signs on the MRV but stenosis/occlusion on the DSP. Sixty segments (12%) showed negative signs on both modalities.

Comparing stenosis with normal vessels, a total of 122 segments were analysed. Thirty-eight of these segments (31%) showed signs of stenosis on both modalities. Fifteen segments (12%) showed stenosis on the MRV but normal signs on the DSP. Seven segments (6%) showed normal signs on the MRV but stenosis on the DSP. Sixty-two segments (61%) showed normal signs on both modalities.

Comparing stenosis with occlusion, a total of 379 segments were analysed. Of these, 308 showed occlusions on both modalities. Twenty-six segments (7%) showed occlusion on the MRV but stenosis on the DSP. Seven segments (2%) showed stenosis on the MRV but occlusion on the DSP. Forty-five segments (12%) showed stenosis on both modalities.

Comparing occlusion with healthy veins, a total of 372 segments were analysed. Of these, 303 segments (81%) showed signs of occlusion on both modalities. Four segments (1%) showed occlusion on the MRV but normal on the DSP. Seven segments (2%) showed typical signs on the MRV but occlusion on the DSP. Fifty-eight segments were normal on both modalities (16%).

The Kappa statistic was calculated as a measure of inter-observer agreement and found an almost perfect agreement for both MRV and DSP between the two radiologists included (ĸ = 0.91 and ĸ = 0.80, respectively); see Table 4 and Table 5.

We also looked at the inter-observer disagreement with the segmental plan and found that the biggest diagnostic challenge was related to the vena cava inferior; see Table 6.

## 4. Discussion

In our study, 123 patients received MRV and DSP, and 496 vein segments were analysed for occlusion, stenosis, or normal appearance. To our knowledge, this is the first study to validate an MR-based technique in a large patient group documenting good sensitivity and specificity. Overall, the results were positive, consistent with MRV and DSP, with high sensitivity, specificity, positive and negative predictive values, and a good inter-observer agreement. However, the negative predictive value for stenosis and occlusion versus normal was only 67%, but this value is considered less clinically important since DVT has been identified beforehand. Moreover, the positive predictive value for stenosis versus normal (72%) and specificity for stenosis versus occlusion (59%) also appear low. These results suggest that there is a tendency to overestimate stenotic areas on MRI, which may be caused by physiological venous contractions and can be confirmed by DSP.

In agreement with our study, a systematic review and meta-analysis from 2015 evaluating the diagnostic accuracy of magnetic resonance venography in the detection of DVT show comparable results with pooled sensitivity and specificity at 93% and 95%, respectively. The study did not distinguish between occlusions and stenosis [16]. A study from 2021 also evaluated the diagnostic accuracy of MRI in the detection of iliac vein obstruction, showing a sensitivity of 96.9%, specificity of 88.9%, positive predictive value of 98.4%, and negative predictive value of 80%. The accuracy was 95.9%. This study graded the occlusion and concluded that it is difficult to differentiate the non-stenotic from the mild level of iliac vein stenosis [17]. As described, our results also suggest some difficulty in differentiating stenosis versus normal veins. Evans et al. found no statistically significant difference between MRI and contrast venography in the detection of DVT. The sensitivity was 100% and the specificity was 95% [18].

Despite the challenges of differentiating stenotic veins from normal veins and the low negative predictive value for stenosis and occlusion versus normal lumen, our results indicate that MRV is a good and valid method in detecting and following patients with DVT in the pelvis. Our institute uses MRV to follow-up patients six months, one year, and two years after the intervention.

There is more than one advantage for MRV versus DSP. Still, a determining factor is that MRV is a non-invasive procedure without ionising radiation and contrast, unlike DSP, which is an invasive procedure using both radiation and contrast. Pelvis radiation is a significant concern, especially in young patients (less than 40 years old) whose reproductive organs are radiosensitive [2], and contrast administration can lead to allergic and nephrotoxic reactions [1,6,19,20]. Furthermore, MRV provides important information about the blood clot itself, whether it is acute or the full extent of the blood clot, and information about peri- and paravascular anatomy, which is essential information when intervention is planned [1,6,21,22,23,24]. As described in the method section, a DVT without diffusion restriction would be excluded because it would be interpreted as a DVT present for more than 14 days. A DSP is positive for DVT when a filling defect or a non-opacification of the vein is seen [10] and provides, therefore, no information about the blood clot itself or peri- and paravascular anatomy.

MRV provides valuable information on peri- and paravascular pathology in patients suspected of DVT. This is especially useful when underlying pathology must be clarified. DVT symptoms can be caused by various underlying conditions, including primarily peripheral deep venous thrombosis, trauma, immobilisation, surgery, malignancy, and the rarer May–Thurner syndrome [25]. MRV can, in some cases, contribute essential knowledge before intervention is planned. Wolpert LM et al. suggest MRI is the best modality for evaluating patients for May–Thurner syndrome [25]. Patients with May–Thurner syndrome often need stents as preventive treatment aimed at preventing the recurrence of DVT.

The MRI images can be reproduced according to our suggested protocol, making introducing this to other radiology departments possible. Because of the apparent advantages of MRV versus DSP, the method should be considered when ilio-femoro-caval DVT is present.

Some studies emphasise that CTV is also a good and valid method for investigating acute and post-thrombotic central venous disease, providing good anatomical detail and accurate thrombus detection. Still, CTV has disadvantages in ionising radiation and contrast administration [2,3,4,5,6].

The disadvantages of using MRI are the cost, time resources, limited availability, interpretation requiring expertise, and the fact that the method cannot be performed in patients with MRI-incompatible devices and patients who have undergone recent surgery [2]. Despite these disadvantages, the advantages of MRV seem to make up for it when DVT in the ilio-femoro-caval veins is suspected, and intervention must be planned.

The experts’ interpretation requirement should not prohibit departments without the expertise, as the MRV procedure can be performed locally, and the images can be interpreted elsewhere, i.e., at departments with the necessary expertise, and where the intervention procedure probably will be performed.

Several limitations were found in this study. First, this study only includes 123 patients (246 image sets and 496 segments), although this study has the largest patient cohort compared with similar studies. Secondly, only two observers were included, which might limit the scope of interpretation diversity. Each observer was blinded to the other’s assessment. If the two observers disagreed, a consensus interpretation of the imaging was made, strengthening the method behind a limited number of observers. This study does not explore a more general limitation of MRI as the optimisation possibilities within the technique used. The main limitation of MRI for diagnosing DVT is the artefacts made by stents or different implants, which influence the image quality.

Further, MRIs are limited by their availability, cost, patients with claustrophobia, pacemakers, or other contraindications. Finally, data are not provided on patient outcomes following diagnosis, which could be necessary to evaluate MRI’s overall clinical utility in DVT management. During follow-up of patients who have undergone catheter-directed thrombolysis, imaging is emphasised rather than clinical findings. The reason to perform imaging-based follow-up is that it allows for reinterventions at an earlier stage than would be the case if reinterventions were only performed based on clinical symptoms such as worsening of the Villalta score. A former study by Madsen et al. did show good clinical results up to 24 months after intervention from a group of patients who were treated according to the described protocol [15].

This study did not validate the ability of the MRV method to distinguish between acute (new) from chronic (old) venous thrombosis. It is our experience that this distinction is most often easy with the acute thrombus causing dilatation of the vein and muscular and subcutaneous swelling of the leg. On the other hand, chronic venous thrombosis is often characterised by stenosis or even a string-like appearance of the vein or web-like structures within the lumen. In clinical MRI today, acute thrombosis is diagnosed by diffusion restriction with high B1000 and through apparent diffusion coefficient (ADC), which were unfortunately not possible in this study due to the method used. For quantification of the diagnostic accuracy of MRV in distinguishing acute from chronic thrombosis, however, a more formal study would need to be performed, including a larger group of patients with chronic stenosis or occlusions than was available for this study.

## 5. Conclusions

The current results showed that MRV is an accurate method to analyse DVT in the pelvic veins. Since there is no use of intravenous contrast, no use of ionising radiation, and with the opportunity to provide anatomical and pathological information around the vessels, the balanced MRI sequence seems to be a good and accurate method to analyse DVT in the pelvic veins. We suggest using MRV as standard procedure when DVT in the ilio-femero-caval veins is suspected and intervention is considered. This paper does not focus on possibilities for optimisation using the full extent of the MRI techniques or include long-term follow-ups in patients where the MRI findings correlate to the risk for re-thrombosis, which could be explored in future research projects.

## Figures and Tables

**Table 1:** Protocol of balanced fast field echo (BFFE) sequences. TE (echo time), TRA (transversal), VCG (vectorcardiogram), SAG (sagittal), COR (coronal), and TR (repetition time).

	Sequences	Echo Time (Milliseconds)	Repetition Time (Milliseconds)
1	Survey	4.6	7.7
2	TRA BFFE VCG	1.67	3.1
3	SAG BFFE VCG	1.67	3.1
4	COR BFFE VCG	1.87	3.7
5	TRA T2w MVDX RT	100	4095

**Table 2:** Patient characteristics.

	Numbers (*n*)	Mean Age (Range)	<20 Years (*n*)	20–40 Years (*n*)	40–60 Years (*n*)	<60 Years (*n*)
Female	80	36.7 (15–72 years)	19	32	18	11
Male	43	46.9 (16–75 years)	6	5	15	17
Total	123	40.3	25	37	33	28

**Table 3:** Summarised results of the analysed segments.

	Positive Predictive Value	Negative Predictive Value	Sensitivity	Specificity	Accuracy
Stenosis and occlusion vs. normal	0.95	0.67	0.93	0.76	0.9
Stenosis vs. normal	0.72	0.9	0.84	0.81	0.82
Stenosis vs. occlusion	0.92	0.84	0.98	0.59	0.91
Occlusion vs. normal	0.99	0.89	0.98	0.94	0.97

**Table 4:** The inter-observer agreement as Kappa statistic for MRV includes Po: proportion of observed agreement, Pe: proportion of chance agreement.

MRV	Observer B+	Observer B−	Total	Po/Pe/Kappa (κ)
Observer A+	403	15	418	0.95
Observer A−	24	417	441	0.50
Total	427	432	859	0.91

**Table 5:** The inter-observer agreement as Kappa statistic for DSP includes Po: proportion of observed agreement, Pe: proportion of chance agreement.

DSP	Observer B+	Observer B−	Total	Po/Pe/Kappa (κ)
Observer A+	375	7	382	0.94
Observer A−	19	64	83	0.72
Total	394	71	465	0.80

**Table 6:** Disagreement. MRV: disagreement between observers on MRV; catheter-directed thrombolysis: disagreement between observers on catheter-directed thrombolysis; Observer 1: different results on different modalities for Observer 1; Observer 2: different results on different modalities for Observer 2.

Anatomical Landmarks	MRV	Catheter-Directed Thrombolysis	Observer 1	Observer 2
Vena iliaca communis dxt.	6	3	4	7
Vena cava inferior	14	7	21	15
Vena femoralis communis sin.	3	3	4	4
Vena iliaca external sin	0	1	2	4
Vena iliaca communis sin.	5	7	7	6
May–Thurner vena iliaca communis	26	0	0	0
May–Thurner vena iliaca externa	10	1	0	0

## Data Availability

The data presented in this study are available on request from the corresponding author.

## References

[1] Carpenter J.P., Holland G.A., Baum R.A., Owen R.S., Carpenter J.T., Cope C. (1993). Magnetic resonance venography for the detection of deep venous thrombosis: Comparison with contrast venography and duplex Doppler ultrasonography. J. Vasc. Surg..

[2] Karande G.Y., Hedgire S.S., Sanchez Y., Baliyan V., Mishra V., Ganguli S., Prabhakar A.M. (2016). Advanced imaging in acute and chronic deep vein thrombosis. Cardiovasc. Diagn. Ther..

[3] Heit J.A., Spencer F.A., White R.H. (2016). The epidemiology of venous thromboembolism. J. Thromb. Thrombolysis.

[4] Aschauer M.A., Keeling I.M., Salvan-Schaschl C.V., Knez I., Binder B., Raggam R.B., Trantina-Yates A.E. (2022). Gadofesveset-Trinatrium-Enhanced MR Angiography and MR Venography in the Diagnosis of Venous Thromboembolic Disease: A Single-Center Cohort Study. Diseases.

[5] Rahaghi F.N., Minhas J.K., Heresi G.A. (2018). Diagnosis of Deep Venous Thrombosis and Pulmonary Embolism: New Imaging Tools and Modalities. Clin. Chest Med..

[6] Silickas J., Black S.A., Phinikaridou A., Gwozdz A.M., Smith A., Saha P. (2018). Use of computed tomography and magnetic resonance imaging in central venous disease. Methodist DeBakey Cardiovasc. J..

[7] Goldhaber S.Z., Magnuson E.A., Chinnakondepalli K.M., Cohen D.J., Vedantham S. (2021). Catheter-directed thrombolysis for deep vein thrombosis: 2021 update. Vasc. Med..

[8] Haig Y., Enden T., Grøtta O., Kløw N.E., Slagsvold C.E., Ghanima W., Sandvik L., Hafsahl G., Holme P.A., Holmen L.O. (2016). Post-thrombotic syndrome after catheter-directed thrombolysis for deep vein thrombosis (CaVenT): 5-year follow-up results of an open-label, randomised controlled trial. Lancet Haematol..

[9] DeWeese J., Rogoff S. (1963). Phlebographic patterns of acute deep venous thrombosis of the leg. Surgery.

[10] De Valois J.C., van Schaik C.C., Verzijlbergen F., van Ramshorst B., Eikelboom B., Meuwissen O. (1990). Contrast venography: From gold standard to ‘golden backup’ in clinically suspected deep vein thrombosis. Eur. J. Radiol..

[11] Özbudak Ö., Eroğulları I., Öğüş C., Çilli A., Türkay M., Özdemir T. (2006). Doppler ultrasonography versus venography in the detection of deep vein thrombosis in patients with pulmonary embolism. J. Thromb. Thrombolysis.

[12] Kim K.A., Choi S.Y., Kim R. (2021). Endovascular Treatment for Lower Extremity Deep Vein Thrombosis: An Overview. Korean J. Radiol..

[13] Tran T.T., Kristiansen C.H., Thomas O., Roy S., Haidl F., Ashraf H., Kløw N.E., Stavem K., Lauritzen P.M. (2022). Indirect CT venography of the lower extremities: Impact of scan delay and patient factors on contrast enhancement and examination quality. Eur. Radiol..

[14] Sampson F.C., Goodacre S.W., Thomas S.M., van Beek E.J. (2007). The accuracy of MRI in diagnosis of suspected deep vein thrombosis: Systemic review and meta-analysis. Eur. Radiol..

[15] Madsen C.P., Gesla J., Vijdea R.L., Serifi M.A., Christensen J.K., Houlind K. (2018). Results of catheter-directed thrombolysis for acute ilio-femoral deep venous thrombosis—A retrospective cohort study. JRSM Cardiovasc. Dis..

[16] Abdalla G., Fawzi Matuk R., Venugopal V., Verde F., Magnuson T., Schweitzer M., Steele K. (2015). The diagnostic accuracy of magnetic resonance venography in the detection of deep venous thrombosis: A systematic review and meta-analysis. Clin. Radiol..

[17] Shi W.-Y., Xue H.-L., Chen L., Gu J.-P. (2021). Non-enhanced multimodal magnetic resonance imaging in assessment of iliac vein obstruction with or without thrombosis. Abdom. Radiol..

[18] Evans A.J., Sostman H.D., Knelson M.H., Spritzer C.E., Newman G.E., Paine S.S., Beam C.A., Evans H.D.S.A.J., Laissy J.P., Cinqualbre A. (1993). Detection of deep venous thrombosis: Prospective comparison of MR imaging with contrast venography. AJR.

[19] Wittens C., Davies A.H., Bækgaard N., Broholm R., Cavezzi A., Chastanet S., de Wolf M., Eggen C., Giannoukas A., Gohel M. (2015). Editor’s Choice—Management of Chronic Venous Disease: Clinical Practice Guidelines of the European Society for Vascular Surgery (ESVS). Eur. J. Vasc. Endovasc. Surg..

[20] Montgomery K.D., Potter H.G., Helfet D.L. (1995). Magnetic resonance venography to evaluate the deep venous system of the pelvis in patients who have an acetabular fracture. J. Bone Surg. Am..

[21] Van Dam L.F., Dronkers C.F.A., Gautam G., Eckerbom Å., Ghanima W., Gleditsch J., von Heijne A., Hofstee H.M.A., Hovens M.M.C., Huisman M.V. (2020). Magnetic resonance imaging for diagnosis of recurrent ipsilateral deep vein thrombosis. Blood.

[22] Fraser D.G., Moody A., Morgan P., Martel A.L., Davidson I. (2002). Diagnosis of lower-limb deep venous thrombosis: A prospective blinded study of magnetic resonance direct thrombus imaging. Ann. Intern. Med..

[23] Kao C.-C., Chen C.-W., Tseng Y.-H., Tsai Y.-H., Wang S.-C., Huang Y.-K. (2020). Non-contrast-enhanced magnetic resonance imaging: Objective figures in differentiation between acute and chronic deep venous thrombosis in the lower extremities. Phlebology.

[24] Chen C.-W., Ting H., Chen P.-Y., Weng J.-C., Hsu Y.-C., Wang S.-C., Tseng Y.-H., Huang Y.-K. (2021). Usefulness of triggered non-contrast-enhanced magnetic resonance angiography in assessing lower extremity venous disease. Medicine.

[25] Wolpert L.M., Rahmani O., Stein B., Gallagher J.J., Drezner A.D. (2002). Magnetic Resonance Venography in the diagnosis and management of May-Thurner syndrome. Vasc. Endovasc. Surg..

